# Simulating the pathway from life history to phylogeny

**DOI:** 10.1111/nph.70881

**Published:** 2026-01-09

**Authors:** Kieran N. Althaus, Andrew L. Hipp

**Affiliations:** ^1^ Herbarium and Center for Tree Science The Morton Arboretum Lisle IL 60532 USA; ^2^ Committee on Evolutionary Biology University of Chicago Chicago IL 60637 USA

**Keywords:** agent‐based simulation, evolutionary rates, longevity, phylogenetic history, substitution rates

## Abstract

This article is a Commentary on Smith *et al*. (2026), **250**: 661–671.

An old oak tree may mate billions of times, produce millions of seeds, and, if it's lucky, leave a few reproductive offspring over the course of centuries to a millennium or more. By the end of its life, it may have witnessed scores of human generations or thousands of *Arabidopsis* generations, each with comparatively few mating opportunities per individual. Variation in longevity and other aspects of life history is reflected in phylogenetic trees through effects on mutation rates (μ), which determine the input of new variation, and effective population sizes ($N_{e}$), which affect the rate at which alleles reach fixation. Mutation and fixation together form the substitution rates that we model in phylogenetic inference, forming a mechanistic link between microevolution, demography, and macroevolution.

*…Smith* et al. *highlight the power of agent‐based simulations to link population‐level demographic processes with macroevolutionary patterns*.

But how exactly do differences in longevity affect evolutionary rates and our ability to reconstruct phylogenetic history? In an article published in this issue of *New Phytologist*, Smith *et al*. (2026; pp. 661–671) develop an agent‐based simulation to tease apart the effects of several life‐history and demographic traits on substitution rate variation and phylogenetic discordance in plants, and then test whether their conclusions hold in an empirical study of four plant clades. Their work builds on research going back to the modern synthesis (Simpson, 1944, p. 137) and extending to the past two decades (e.g. Smith & Donoghue, 2008; Bromham, 2009; Beaulieu *et al*., 2013; Lanfear *et al*., 2013) on how demography and microevolution influence evolutionary rates. A key insight of work to date is that demographic processes – such as lifespan, population growth rate, and generation time – alter both the effective population size and mutation rate of a population (Waples, 2016), although these effects on genetic divergence within species do not invariably drive speciation rates (Singhal *et al*., 2022). One thing that is unclear is which aspects of demography most strongly influence the relationship between longevity and substitution rates.

Smith *et al*. simulate populations of diploid organisms that vary (among but not within simulations) in maximum age, death rate, age at maturity, population carrying capacity, and somatic and meiotic mutation rates (Fig. 1). Each starting population diverges twice in the course of each simulation to yield a three‐tip phylogeny. Substitution rates are not directly adjusted, but arise from the origins, inheritance, and fixation of genomic variation among independently evolving branches on each organism (the latter being a potentially important if under‐recognized source of adaptive genetic variation; Xian *et al*., 2025). Thus, substitution rates are an emergent property of the life‐history parameters of all the individuals comprising each simulated phylogeny. DNA sequences of the individuals living at the end of each simulation are used to infer phylogeny and calculate sequence diversity. The authors then compare their simulation results to empirical phylogenetic and life‐history data for three major angiosperm clades and the gymnosperms to assess whether the simulated patterns of discordance and substitution rates match those found in natural systems.

**Fig. 1:**
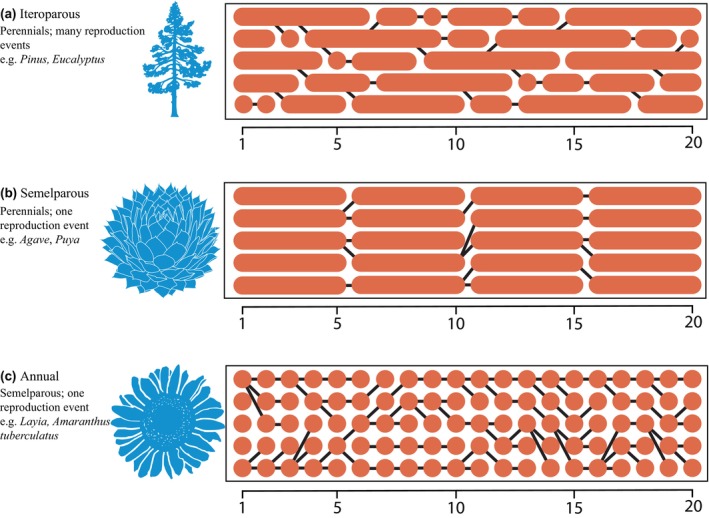
Simulating the pathway from life history to branching history. The agent‐based simulation developed by Smith *et al*. (2026; pp. 661–671, in this issue of *New Phytologist*) enables their investigation of how age to first reproduction, death rate, maximum age, carrying capacity, and mutation rates influence substitution rates and phylogenetic discordance. While previous work has investigated the effects of longevity on substitution rates (illustrated here, where average longevity decreases from B to A to C), the simulation approach taken here allows the authors to potentially tease apart the effects of mean longevity from overlapping generations (illustrated here, where only A exhibits overlapping generations). All images are from Phylopic.org and free to use under Creative Commons Zero Deed (License: CC0 1.0).

The simulation results largely validate earlier work by Smith & Donoghue (2008), showing that lineages of long‐lived organisms exhibit lower substitution rates than lineages of short‐lived organisms. Because numerous traits covary with lifespan, the mechanisms underlying the correlations in this previous work were unclear. The current study endeavors to tease apart which particular aspects of lifespan influence substitution rates and phylogenetic discordance. The main effects, it turns out, are death rate and maximum age, more than age to first reproduction or mutation rates. Lineages with longer average lifespans have lower substitution rates and higher phylogenetic discordance, supporting earlier analytical work showing that lifespan influences substitution rates through its effect on mean generation time, which is not expected to be strongly correlated with age to first reproduction, particularly in long‐lived organisms (Lehtonen & Lanfear, 2014).

The empirical results corroborate the simulations: Lifespan across four clades is positively correlated with phylogenetic discordance at internal nodes and negatively correlated with substitution rates (reconstructed branch lengths). The difference is particularly striking in the contrast between the woody‐plant clade Fagales, containing beeches, birches, walnuts, and related families, and its sister clade, Cucurbitales, which comprises of a mix of woody and herbaceous species, including annuals, such as pumpkins (*Cucurbita pepo*) and other gourds. Fagales have substantially higher phylogenetic discordance than Cucurbitales, with conspicuously shorter branches.

There are a few possible next steps for this work. One is testing the mechanisms underlying lifespan‐substitution rate correlations across a wider range of phylogenetic scales and natural systems. While the framing and discussion in this paper are focused in part on trees, the mechanisms generating heterogeneity of substitution rates should apply equally to any long‐lived plants, whether that 300‐yr‐old plant is *Silene acaulis*, *Larrea tridentata* or *Quercus robur*. Instraspecific variation might also have a strong effect. *Streptanthus tortuosus* and *Mimulus guttatus*, for example, are polymorphic for annual and perennial life histories, offering natural experiments where longevity varies among individuals and populations within species. Beyond plants, mayflies (order Ephemeroptera) range in lifespan from days to years, and cicadas (superfamily Cicadoidea) may live for a year or for 17 yrs depending on the species. Mammals range in lifespan from less than a year in the wild for Mueller's giant Sunda rat to more than 200 yrs for the bowhead whale. Not surprisingly, there is a known generation time effect on both synonymous and nonsynonymous substitution rates in invertebrates, and synonymous substitution rates in vertebrates (Thomas *et al*., 2010 and references therein). Modeling life‐history differences across clades using Smith *et al*.'s approach could help illuminate whether the demographic constraints on substitution rates found for plants are universal or contingent on body plan, metabolic strategy, size, or reproductive strategy.

Additionally, a closer investigation of overlap in generations and effective population size might provide insights into how the findings in the current study relate to previous work. Smith *et al*. suggest that the effects they observe are due largely to the maintenance of higher ancestral polymorphism within age‐structured populations. They attribute this to overlapping generations in long‐lived organisms. Lower death rates and higher maximum age lead to greater age variance in the population, allowing long‐lived individuals to continually reintroduce old alleles to the population via new offspring (as argued to different ends in Cannon *et al*., 2022). In this way, they argue, longevity mimics population size, leading to higher incomplete lineage sorting. But retention of old alleles in populations with overlapping generations may in fact result in lower effective population sizes relative to populations with discrete generations (Nunney, 1993; Rodrigo & Felsenstein, 1999). Moreover, substitution rates at neutral nucleotide positions are expected to be invariant with respect to effective population size, as the increase in accumulation of mutations with larger numbers of individuals counterbalances the increased time to fixation (Lanfear *et al*., 2014). The analyses presented in the paper may actually reflect the fact that longevity (due either to maximum lifespan or reduced turnover) correlates with mean generation time (Lehtonen & Lanfear, 2014). Further investigation of the simulations conducted, as well as incorporation of selection, might help illuminate whether the findings presented here counter theoretical expectations or are best interpreted in light of those expectations.

Smith *et al*.'s work has practical implications for phylogenetic inference. Heterogeneous molecular rates can systematically bias phylogenetic divergence estimates (Beaulieu *et al*., 2015), leading to false positives in diversification rate shift tests (Shafir *et al*., 2020). Smith *et al*.'s approach may provide a framework for predicting the conditions under which shifts in diversification rates will be most strongly biased. Moreover, the effects of life‐history traits on macroevolutionary patterns and rates of molecular evolution extend well beyond the phylogenetic effects studied here. Many species are already maladapted, and climate change is expected to worsen this problem (Brady *et al*., 2019). Long‐lived populations are expected to evolve in response to changing adaptive optima with greater lag than short‐lived populations (Zeineddine & Jansen, 2009) by the same mechanisms that constrain substitution rates, though with the potential for higher resilience under fluctuating selective regimes (Cannon *et al*., 2022). The simulation method presented in the current study might as a consequence be adaptable to a wider range of questions in macroevolution.

Beyond their findings about longevity, Smith *et al*. highlight the power of agent‐based simulations to link population‐level demographic processes with macroevolutionary patterns. Whether and how patterns observed at these two scales are related is a long‐standing question in evolutionary biology (Schluter, 2024). Smith *et al*.'s simulation framework complements analytical approaches to teasing apart the effects of individual life‐history traits on phylogenetic patterns. Their approach has the potential to substantially advance our understanding of how the lives of individual organisms and their interactions as populations scale up to shape macroevolution.

## Disclaimer

The New Phytologist Foundation remains neutral with regard to jurisdictional claims in maps and in any institutional affiliations.
